# Pediatric pseudo-tumor of the ischio-pubic ramus in virtual non-calcium photon-counting scanner: A case report

**DOI:** 10.1016/j.radcr.2026.03.061

**Published:** 2026-04-23

**Authors:** Clara Corelli, Thomas Balligand, Roberto Rodriguez, Marion Hamard, Sana Boudabbous

**Affiliations:** Division of Radiology, Department of Diagnosis, Geneva University Hospitals, Geneva, Switzerland

**Keywords:** Pseudo-tumor, Van Neck-Odelberg disease, Photon-counting, Virtual non calcium, Low dose, Ischiopubic synchondrosis

## Abstract

We report a case of ischio-pubic ramus synchondrosis in an 11-year-old elite athlete initially misinterpreted as a tumor on MRI. We aim with this case to highlight the role of the advanced technique Virtual Non-Calcium imaging in photon-counting CT as a new low-dose imaging modality with straightforward interpretation, even for junior radiologists.

## Introduction

Van Neck–Odelberg disease is a benign, self-limiting condition characterized by osteochondritis of the ischiopubic synchondrosis, typically observed in children during skeletal maturation. It results from asymmetric mechanical stress at the ischiopubic synchondrosis, leading to inflammation and delayed ossification [[1], [2], [3]]. Because MRI findings may mimic tumor or infection, diagnosis can be challenging. The role of photon-counting CT, particularly virtual non-calcium imaging, in improving diagnostic confidence in this entity remains insufficiently reported.

## Case report

**Fig. 1:**
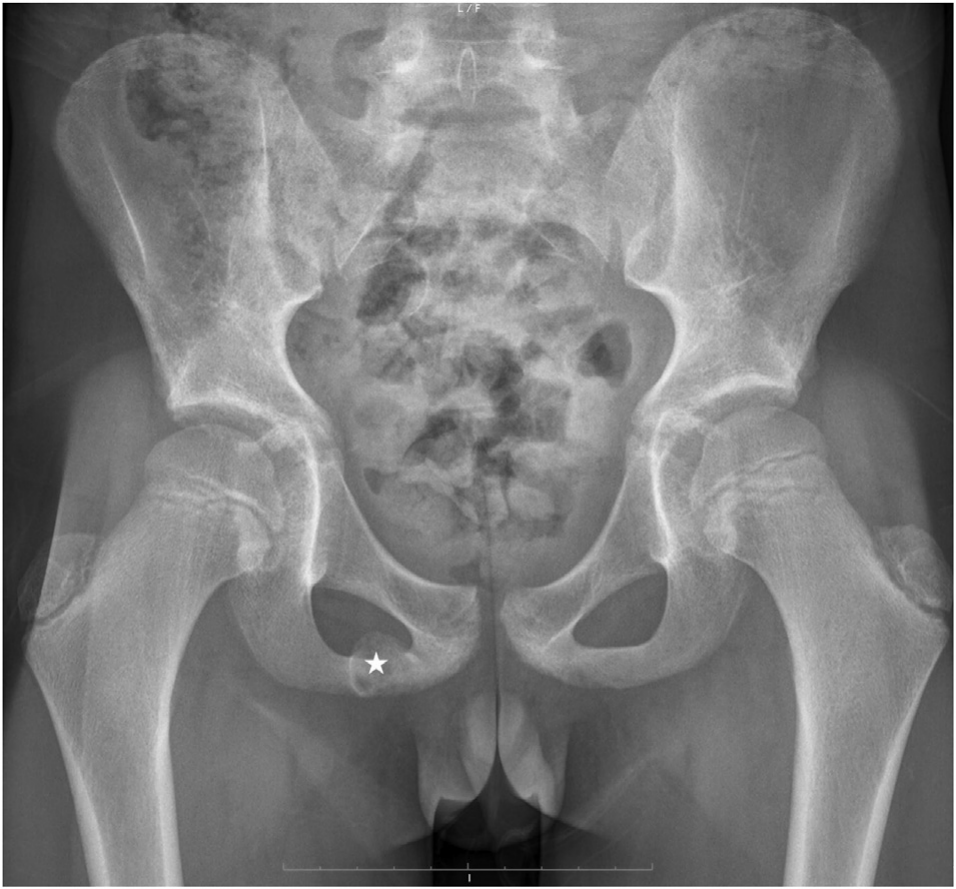
Pelvic radiograph showing a heterogeneous, expansile intraosseous lesion of the right ischiopubic ramus (asterisk).

**Fig. 2:**
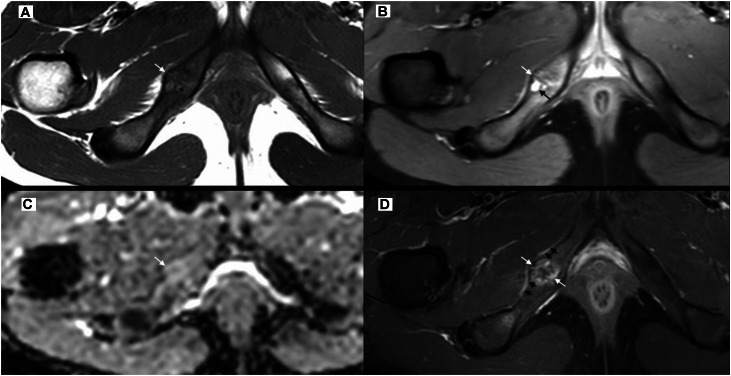
(A) Axial T1, (B) Axial PDFS, (C) ADC, (D) Post contrast T1 fat sat: images showing a T1 isointense, PDFS cystic areas hyperintenses (black arrow in B), without restriction of diffusion (white arrow in C), with peripheral pseudo lobular contrast enhancement after injection (white arrows in D). Note the presence of periosteal oedema without mass (short black arrows in D).

**Fig. 3:**
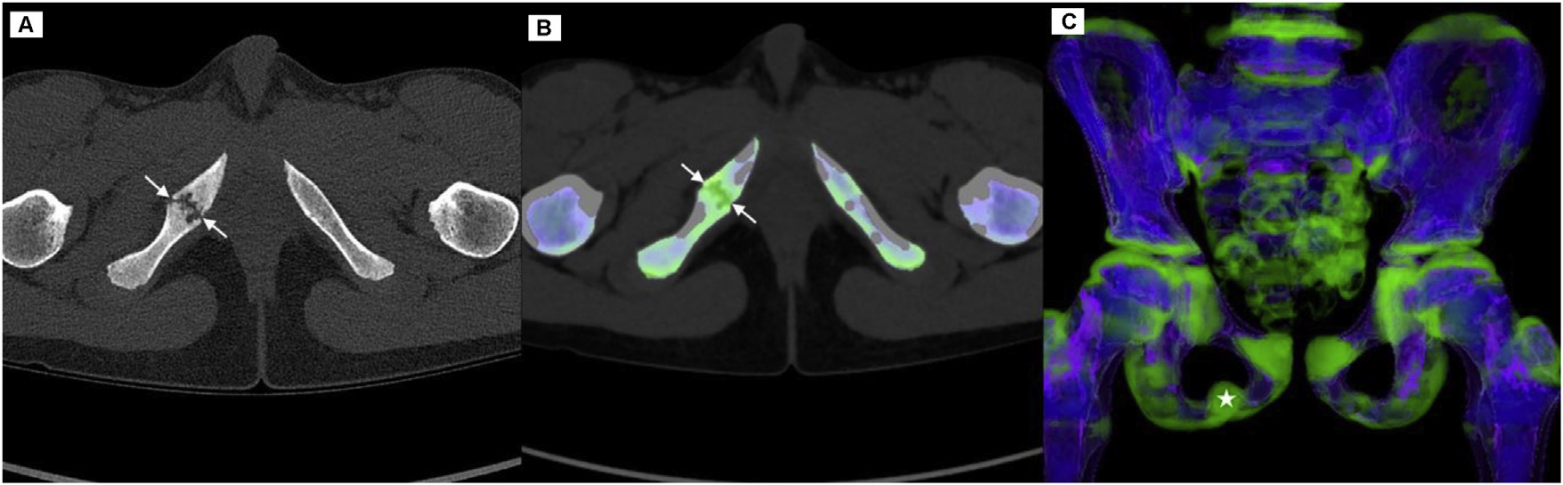
(A) Axial PC-CT (B-C) Axial and coronal PC-CT Q-Peak protocol images with VNC reconstruction showing edema centered on the synchondrosis corresponding to the enhanced lesion in MRI (white arrows in B). Note in the 3D rendering of VNC coronal reconstruction, the obvious asymmetry compared to contralateral branch (asterisk).

An 11-year-old elite-level handball player, with no relevant medical or surgical history and otherwise in good health, presented with low back and buttock pain radiating to the right knee and calf. The clinical picture was consistent with right-sided sciatica. Due to the persistence of symptoms and their functional impact, a pelvic radiograph was performed (Fig. 1), revealing a hypertrophic bone involving the right ischiopubic ramus with pseudo-lucency, peripheric sclerosis and abrupt transition to adjacent bone. No cortical lysis or specific matrix was evident. Pelvic magnetic resonance imaging (MRI) (Fig. 2) was performed the following day and revealed a centrally located intramedullary lesion of the right ischiopubic ramus, measuring 18 × 16 mm on axial images. The lesion was well defined, with sclerotic margins and showed no pathological fracture or cortical breach. MRI signal characteristics were heterogeneous, with cystic areas appearing hyperintense on proton density (PD) fat-saturated sequences, isointense on T1-weighted images and showing peripheral enhancement after gadolinium injection. The patient was referred to musculoskeletal radiology specialist, where a photon-counting CT scan with VNC technique was performed in very low dose (113 mGy.cm^2^) (Fig. 3). The Table 1 summarized protocol parameters of CT acquisition realized in Alpha-pro Siemens Naeotom^R^ scanner (Germany). These morphological images showed enlargement of the right ischiopubic synchondrosis with multiple para-articular cysts surrounded by peripheral sclerosis without occupying process, thus suggesting the diagnosis of ischiopubic synchondrosis. A specific protocol named Q peak (Quantum peak) allowing VNC reconstruction was performed. It revealed edema in the bone surrounding the lesion without extension, supporting the benign and nonaggressive nature of the lesion. The diagnosis of Van Neck–Odelberg disease was ultimately established with confidence.

**Table 1:** Summary of the acquisition parameters for the PCCT Q-Peak protocol

CT type	SIEMENS Naeotom alpha.pro Mutli CT energy acquisition
Acquisition dose	113 mGy.cm
Type of acquisition	Q Peak UHR
CARE dose et CARE kV	On
kV	Sn 150/90
Reference mAs	161/143
Effective mAs	59/80
Reconstruction Kernel	Bone
Pitch	0.6
Rotation time	0.25 s
Tilt	No
Slice thickness	0.2mm
Kernel	Br40
Iterative reconstruction	QIR3

The patient was managed conservatively with symptomatic treatment and tempory reduction of sports activity. Progressive clinical improvement was observed, followed by complete resolution of the pain. The patient subsequently returned to handball without recurrence of the symptoms. Additional imaging was avoided given the benign diagnosis and clinical follow-up alone was recommended as no correlation is expected between imaging and clinical findings.

## Discussion

A total of 22 case reports were identified in the literature (Table 2), including one case series of 21 cases, accounting for a total of 45 documented patients with Van Neck–Odelberg disease. The reported age range was between 4 and 15 years, with one exceptional case reported at 88 years of age [4].

**Table 2:** Review of the literature identified published case reports of van Neck-Odelberg disease

Year of publication	Author	Article type	Number of cases	Ages of cases (years)	Sex	Imaging modalities *(listed in chronological order)*	Diagnosis
2010	Oliveria [7]	Case report	1	8	Male	X-ray, Bone scintigraphy, CT, MRI	Benign lesion
2016	Beyitler [8]	Case report	1	7	Male	X-ray, MRI	Subacute fracture
2017	Chaudhari [9]	Case report	1	12	Male	X-ray, CT, MRI	*Not described*
2017	Morante [1]	Case report	1	8	Male	X-ray, MRI	VNOD
2018	Pirimoglu and Sade [10]	Case report	1	8	Male	X-ray, MRI	VNOD
2020	Ceri and Sperati [11]	Case report	1	8	Male	X-ray, MRI	VNOD
2020	Walter [12]	Case report	1	10	Male	X-ray, MRI	Pathological fracture
2021	Macarini [13]	Case series	2	8 ;12	Male ;Male	1-2 : X-ray, MRI, CT	*Not described*
2021	Narayanan [14]	Case report	1	11	Male	X-ray, MRI	After X-ray : Pathological fracture / infection After MRI: VNOD
2021	Sabir [15]	Case report	1	14	Male	X-ray, MRI	VNOD
2021	Schneider [16]	Case series	21	8-13	13 males, 8 females	X-ray, CT, MRI	9 : Bone tumor 3 : Fracture 2 : Osteomyelitis 1 : Bone metastases 6 : VNOD
2021	Tam [17]	Case report	1	12	Male	X-ray, MRI	VNOD
2022	Camacho [2]	Case report	1	15	Male	X-ray, MRI	After X-ray and MRI : Pathological fracture Add bone scintigraphy : VNOD
2022	Fonseca [5]	Case report	1	6	Male	X-ray	VNOD
2022	Korkmazer [18]	Case series	2	10, 7	Female	1 : X-ray, CT, MRI 2 : X-ray, MRI	1 : Benign lesion 2 : VNOD
2022	Moreira [3]	Case report	1	4	Female	X-ray, MRI	VNOD
2023	Hursoy [19]	Case series	2	8, 4	Female, male	1, 2 : MRI	*Not described*
2024	Afaque [20]	Case report	1	13	Female	X-ray, MRI	VNOD
2024	Mcloughlin [21]	Case report	1	9	Female	X-ray, MRI	*Not described*
2024	Wang [4]	Case report	1	88	Female	X-ray	VNOD
2024	Nagaraj [6]	Case report	1	11	Male	X-ray	Benign lesion
2025	Krug [22]	Case report	1	7	Male	X-ray, MRI	Benign lesion

In all published cases, initial imaging included standard radiography, which consistently revealed suspicious findings prompting further diagnostic evaluation. In 39 out of 45 cases, MRI was performed, whereas only 3 publications reported stopping investigations at plain radiographs [[4], [5], [6]]. This pattern highlights the diagnostic challenge of relying solely on radiographic findings. MRI played a critical role in establishing the diagnosis, consistently demonstrating characteristic features: lesions that were isointense on T1-weighted images, showed peripheral enhancement after gadolinium administration, and were consistently located at the ischiopubic synchondrosis. However, in non-expert center and with novice radiologists, MRI could be challenging for the diagnosis [[7], [22]].

In less than half of the reported cases, additional imaging was performed using CT. However, as is well recognized, CT is not the preferred modality in the pediatric population due to their increased sensitivity to ionizing radiation [23]. In our case, the utility of PCCT was discussed. PCCT offers the advantage of high spatial resolution while significantly reducing radiation dose [24] (eg, dose reduction of 43% and up to 70% in children under 6 years of age in pediatrics temporal bone CT using PCCT vs dual-energy CT [25]), which may allow for improved diagnostic confidence in select cases where MRI findings remain equivocal. In our case, the PCCT examination performed with a dual-source protocol resulted in an effective dose of 1.61 mSv. For comparison, the effective dose of a standard anteroposterior pelvic radiograph is approximately 0.26 mSv, yielding a ratio of 6.19. This means that the effective dose delivered by our PCCT corresponds to that of about 6 pelvic radiographs**,** without missing that in majority of cases 2 incidences at least are performed.

PCCT is an emerging imaging modality that offers novel technical advantages. Unlike conventional dual-energy CT, PCCT directly converts X-ray photons into electrical signals using semiconductor detectors, allowing for improved spatial resolution and spectral imaging [26].

As of mid-2025, a PubMed search revealed 27 published articles specifically addressing the use of PCCT in musculoskeletal imaging, highlighting a growing interest in this field [[27], [28], [29], [30], [31], [32], [33], [34], [35], [36], [37], [38], [39], [40], [41], [42], [43], [44], [45], [46], [47], [48], [49], [50], [51], [52], [53]].

In our case, PCCT allowed, in addition to the morphological assessment of the bone, the analysis of medullary oedema and its extension, which corresponded to MRI findings. This technique, used in adults for bone fracture dating (acute vs ancient), is sensitive to medullary bone conversion by subtracting the mineralized components of bone. The Q-Peak technique by Siemens^R^, integrated into the PCCT system, leverages advanced photon-counting detectors to achieve high spectral resolution. This enables the precise differentiation between bone and soft tissues by selectively eliminating the calcified bone components, thus generating VNC images. These VNC images are particularly useful for assessing soft tissue changes such as bone marrow oedema [54].

This technique by summing excellent bone analysis and detection of oedema is promising. Moreover, new PCCT permits a low dose opening interesting perspectives to pediatric indications such osteomyelitis and bone fractures with non-conclusive radiographs.

## Conclusion

This case highlights the diagnostic utility of photon-counting CT in evaluating the ischiopubic ramus synchondrosis.

This imaging modality provides high-resolution anatomical information with low radiation exposure, which is particularly important in the pediatric population.

In this case, detecting oedema is an added value to high resolution CT; facilitating differentiation between benign conditions such as Van Neck–Odelberg disease and other diagnoses including pathological fractures or malignant lesions. This case supports a potential complementary role for photon-counting CT in selected pediatric cases when MRI findings are equivocal or unavailable.

## Patient consent

Written informed consent for the publication of this case report was obtained from the guardian of the patient (mother).
